# Implementing an initiative promote evidence-informed practice: part 2—healthcare professionals’ perspectives of the evidence rounds programme

**DOI:** 10.1186/s12909-019-1488-z

**Published:** 2019-03-06

**Authors:** Aislinn Conway, Maura Dowling, Declan Devane

**Affiliations:** 1 Health Research Board Trials Methodology Research Network, School of Nursing and Midwifery, National University of Ireland Galway, Galway, Ireland; 2 School of Nursing and Midwifery, National University of Ireland Galway, Galway, Ireland

**Keywords:** Dissemination, Implementation science, Knowledge translation, Evidence-informed practice, Health services research, Barriers, Facilitators, Sustainability, Focus groups, Interviews

## Abstract

**Background:**

The translation of research into clinical practice is a key component of evidence-informed decision making. We implemented a multi-component dissemination and implementation strategy for healthcare professionals (HCPs) called Evidence Rounds. We report the findings of focus groups and interviews with HCPs to explore their perceptions of Evidence Rounds and help inform the implementation of future similar initiatives. This is the second paper in a two-part series.

**Methods:**

We employed total population, purposive sampling by targeting all of the health care professionals who attended or presented at group sessions exploring the evidence on clinical questions or topics chosen and presented by the HCPs. We conducted and audio-recorded in-person focus groups and one-to-one interviews, which were then transcribed verbatim. Two authors independently coded transcripts. NVivo software was used to collate the primary data and codes. We analysed data guided by the five steps involved in framework analysis; 1) familiarization 2) identifying a thematic framework 3) indexing 4) charting 5) mapping and interpretation.

**Results:**

Thirteen HCPs participated, of which 6 were medical doctors an d 7 were nursing or midwifery staff. We identified the following key domains; organisational readiness for change, barriers and facilitators to attendance, barriers and facilitators to presenting, communication and dissemination of information, and sustainability. During focus groups and interviews HCPs reported that Evidence Rounds had a positive impact on their continuing education and clinical practice. They also provided insights into how future initiatives could be optimised to support and enable them to narrow the gap between research evidence and practice.

**Conclusions:**

Individual, departmental and organisational level contextual factors can play a major role in implementation within complex health services. HCPs highlighted how in combination with clinical guideline development, implementation of evidence could be increased. Further research after a longer period of implementation could investigate how initiatives might be optimised to promote the uptake of evidence, improve implementation and expedite behaviour change.

**Electronic supplementary material:**

The online version of this article (10.1186/s12909-019-1488-z) contains supplementary material, which is available to authorized users.

## Background

Evidence-informed decision making is fundamental to the provision of healthcare and central to this is the translation of research evidence into clinical practice. The use of the term “evidence-informed” highlights the need to acknowledge and address contextual influences and consider how the best available evidence can be used in specific circumstances [1].

There is a need to improve translation of research evidence into practice [2]. The ever-growing volume of research publications [3, 4], the complex nature of research [5], gaps in skills [6] such as knowledge of how to interpret statistical information, publication bias [7] and nonlinear, non-rational processes in decision making [8] are just some of the potential barriers to translating evidence into practice. Research is growing in fields that attempt to tackle and narrow the gap between knowledge and action such as knowledge translation (KT), dissemination and implementation science, knowledge mobilisation and knowledge brokering.

KT strategies can employ single or multiple components such as professional educational meetings eg. journal clubs, educational materials, educational outreach visits, knowledge brokers, audit and feedback etc. A limitation of the traditional educational model of journal club is that its primary focus is on the critical appraisal of a single source [9]. A Cochrane systematic review of 81 trials involving nearly 11,000 healthcare professionals found that standalone continuing education meetings and those with additional components can lead to small improvements in patient care and clinical practice with the exception of very complex behaviours [10]. In a systematic review by Giguère and colleagues, printed educational materials appeared to positively effect professional practice outcomes. However, it was not possible to measure the size of the effect in relation to patient outcomes [11]. In another systematic review, there was a lack of evidence to assess the effectiveness of knowledge brokers [12]. A Cochrane review reported that small but important changes to clinical practice can result from audit and feedback [13]. A Cochrane systematic review by Forsetlund and colleagues found moderate quality evidence that for HCPs working in primary and secondary healthcare settings, higher attendance at educational meetings was effective at increasing compliance with a target practice. Interestingly, they found decreased effectiveness for outcomes with a lower level of severity and no evidence of effectiveness for complex behaviours. They recommended the use of strategies to increase attendance although they did not specify the necessary components of these strategies [10].

While outcome evaluations tell us whether an implementation programme does or does not work, they can ignore confounding contextual factors [14] and fail to tell us more about why, how or under what circumstances a programme does or does not work [15]. In a given population, there needs to be an understanding of barriers and facilitators to evidence based practice [16]. To address these issues, it is necessary to examine the process and context. Translation of knowledge is a context-dependent process, contingent on many factors and takes place in complex healthcare systems [17].

While a number of definitions for the term dissemination have been suggested, in this paper we define it as “an active approach of spreading evidence-based interventions to the target audience via determined channels using planned strategies” [18]. McCormack 2013 identified the broad goals of dissemination to clinicians as increasing the reach of the evidence b) increasing the motivation to utilise and apply evidence and c) increasing the ability to utilise and apply evidence [19].

We utilised a multi-faceted KT strategy to actively disseminate evidence to healthcare professionals and promote evidence informed practice including implementation of evidence where appropriate [20]. Our initiative called Evidence Rounds, took place over nine months from July 2016 to March 2017 and featured three core components: 1) six educational group sessions examining the evidence on clinical topics or questions chosen by our target audience, 2) support from a KT professional and 3) the use of multiple modes of delivery to communicate and disseminate information including a dedicated website. For each session, three HCPs presented the evidence on a single topic that was agreed upon by the larger group. It was not mandatory for staff to give presentations rather, they were invited to volunteer to present. In some cases individuals were recommended by their peers and invited by the implementation team to present. After the presentations a discussion forum would take place where the applicability of the evidence was explored and if appropriate, potential resulting actions were identified and discussed by attendees. For example, staff identified a gap in their knowledge relating to evidence around antenatal steroid use for preterm deliveries less than 37 weeks gestational age. So, this topic was chosen to be the focus of an Evidence Rounds educational session. As a result of the Evidence Rounds session, awareness of the evidence increased and it was deemed appropriate to implement the evidence. Further discussed at a multidisciplinary team meeting and other meetings contributed to the process of implementation. The local guideline on preterm premature rupture of the membranes (PPROM) was updated to recommend that antenatal steroids be considered by the consultant dealing with the patient when there is a risk of preterm birth at a gestational age of 23 weeks + 0 days to 23 weeks + 6 days (previously 24 weeks + 0 days). Therefore, this Evidence Rounds educational session led to a guideline update and a change in practice. Some of the core elements of Evidence Rounds were based on Evidence in Practice Groups established by Jacqui LeMay and run by the Clinical Evidence Based Information Service (CEBIS) at University Hospitals Coventry and Warwickshire NHS Trust. We used collaborative processes to design and develop the initiative and actively sought feedback from key stakeholders (HCPs) throughout these phases. By doing this, we could adjust components to better suit the local context and meet the needs and preferences of our target audience. A more comprehensive description of Evidence Rounds and its process of implementation is available in paper 1 of this two-part series [21].

The objectives of this study were to use focus groups and interviews a) to identify HCP-reported barriers and facilitators to attending and presenting at educational group sessions b) to explore HCPs’ views of Evidence Rounds, particularly as a dissemination strategy, and c) to generate insights to improve the sustainability of future initiatives because evidence about the sustainability of KT interventions is still lacking [22, 23].

## Methods

### Study design, setting and participants

We utilised a qualitative study design which can provide valuable insights into contextual factors and intervention features which influence the success of KT interventions [24]. We used total population, purposive sampling and invited all healthcare professionals working in the maternity unit of one urban hospital in Ireland who attended or presented at at least one Evidence Rounds educational session to participate. We excluded students on placement and other attendees who were not employed as health care staff at the hospital because the primary target audience of the initiative was HCPs who attended and presented at group sessions and we were specifically interested in learning more about their perceptions. We did not prespecify a target sample size before recruitment because we expected it to depend on attendance levels, availability and willingness to participate in the study as well as other potential factors. We decided that it was not appropriate to use other studies to provide the required estimates in our population. Nevertheless, focus groups were expected to consist of 4 to 8 participants each. No more than 10 individuals would be interviewed on a 1:1 format. If more than 10 individuals were to volunteer, selection would be prioritised using the following criteria: a) first priority would be given to any attendee type who is under-represented in the focus groups and b) second priority will be given to attendees who volunteered on a first come, first served basis.

### Procedure

#### Focus groups and interviews

We gave potential participants the choice to take part in either a focus group or an interview according to their individual preferences. We displayed posters in areas frequently accessed by our target population. To enhance recruitment, we entered each participant into a draw to win a voucher for a local restaurant. We developed an interview guide around the aims of the study (Additional file 1). We asked participants about the determinants impacting their choice to attend or present at our group sessions and how our initiative performed in relation to the goals identified by McCormack (2013). We questioned them about the sustainability of Evidence Rounds and the factors that might increase the likelihood of sustainability for other initiatives. We asked participants questions about specific components and modes of delivery to find out what worked and did not work for them. Our study was granted ethical approval by the Galway University Hospitals Clinical Research Ethics Committee (CREC). During recruitment, we distributed informed consent packs incorporating a participant information leaflet and consent form (Additional file 2), which all participants read and signed before taking part. We changed potential identifiers to protect the anonymity of our participants.

### Data collection and analysis

We audio-recorded interviews and one author moderated all focus groups and interviews for consistency. Audio files were transcribed *verbatim* by a professional from a transcription service who had signed a confidentiality agreement. We chose to analyse the data using Richie and Spencer’s framework analysis which can be used in applied qualitative research [25]. Our decision was based on its suitability for dealing with focus group and interview data and its focus on prospective actionable outcomes. We utilised an iterative rather than a linear process to complete the five components of this method of analysis:

1. Familiarization

AC who had been present at all recordings re-listened and where appropriate, made corrections and where possible, filled in sections of speech that were inaudible to the transcriber. Two authors (AC and MD) listened to the audio files while reading the corrected transcripts. AC reviewed the observational notes collected by the assistant moderator during the focus groups. Two authors (AC and MD) independently coded the transcripts and noted key points, repeated themes and issues considered important by participants.

2.) Identifying a thematic framework

We began to create a thematic framework drawing from a list of 54 a priori key issues deemed relevant to our study aims, 21 additional emergent issues based from participant responses, and began to connect and look for patterns in participant responses to form analytical themes. The thematic framework took several iterations.

3.) Indexing

We uploaded the transcripts to NVivo Version 11 and systematically applied the thematic framework by assigning nodes and sub-nodes to text within each transcript. As is common in framework analysis papers, some text was coded into multiple nodes [26], others were merged and throughout this stage, we made further refinements to the framework.

4.) Charting

We reviewed the data and made a decision to chart by core themes rather than cases. We created five tables each with a unique domain and used themes, sub-themes and illustrative quotes that demonstrated the range of participant responses. All authors reviewed the tables and made revisions to improve the presentation of data.

5.) Mapping and interpretation

We referred to the main aims of the study and reviewed the tables. We considered the nature and range of participant perspectives. Using this method, it was possible to extract key dimensions of the barriers and facilitators to attending and presenting at Evidence Rounds, their perspectives of our dissemination strategy, and suggestions to make future initiatives more sustainable.

## Results

Thirteen HCPs participated in three focus groups (of between two and four participants), and five in one-to-one interviews. Six medical doctors and seven nursing or midwifery staff participated, of which four were male and nine were female. Our data analysis revealed five core domains regarding HCPs perspectives of Evidence Rounds: (1) barriers and facilitators to attending; (2) barriers and facilitators to presenting; (3) organisational readiness for change; (4) communication and dissemination of information; and (5) sustainability.

### Barriers and facilitators to attendance

This domain included three themes namely; departmental context and resources, social context and individual level factors. Our study demonstrated that attendance levels at Evidence Rounds were determined by the availability and workloads of staff, the organisational climate, the attendance of others (colleagues and senior-level staff), level of interest in the topic and extrinsic factors such as certificates of attendance and continuing education units from a professional body. HCPs who had control over the timing for their daily activities experienced less scheduling-related restrictions compared to those who were providing front-line care on hospital wards. Lunchtime was identified as the most likely time to suit the majority of people. The provision of food and beverages was a facilitator to attendance especially for HCPs who would not get another opportunity to eat during their work shift. Keeping sessions within the advertised timeframe was appreciated by busy HCPs. A number of staff came into work on their days off to attend Evidence Rounds. Some line managers agreed to allow time in lieu for these staff. However, this was not offered to all employees and in general, being off duty was a barrier to attendance. We also identified a previously unknown scheduling conflict with a lunchtime meeting for obstetric staff. This may contribute to the fact that there were fewer attendees from this department. Busy workloads and inadequate staffing levels were barriers to HCPs attending sessions. Understandably, clinical care took priority and staff reported that some colleagues had trouble attending even mandatory training sessions (attendance at Evidence Rounds was voluntary).

All staff viewed the interprofessional and multiple disciplinary nature of Evidence Rounds as a facilitator to their attendance. Teamwork and the breaking down of professional silos were among the positive effects they saw from this approach. Consultant attendance and management support for Evidence Rounds was mentioned repeatedly as having a positive effect on non-consultant hospital doctor (NCHD), nursing and midwifery staff attendance. Senior staff acknowledged that their attendance set an example for junior staff. Some HCPs were motivated by a self-perceived benefit to attending e.g., obtaining professional credits for attendance, certificates of attendance or participation, claiming back time spent or enjoying a free lunch Table 1.

**Table 1:** Themes and sub-themes likely to explain responses to questions about barriers and facilitators to attendance

Theme	Sub-theme	Sample quote	Participant
Departmental context and resources	Scheduling and rostering	“if you can manage your own diaries, I don’t think it makes a big difference to you because if I did attend I could go for lunch afterwards. Whereas a staff member on the ward I think that’s a lot more important to them, that they’re able to get their lunch as well.”	Nurse/Midwife G
		“And there’s no good time in maternity, as far as I could see for any education sessions like this. And it’s an ongoing battle really as to what is the most suitable one. But I’d say perhaps it is as suitable as any time.”	Nurse/Midwife A
		“it kept to the time limit. And I think that’s really important because sometimes things can go way beyond the time frame. And people lose interest. And very often they have other things and deadlines to get to and meetings to get to.”	Physician E
		“And we did ensure, it was one of the things that I did, that staff would get time back and let them know that if they did come in on their time, in their own time they get 2 h’ time away. One or two did come in in their own time but not not [sic] much.”	Nurse/Midwife E
		“the obstetric site has few people turn up, it’s also they have their Friday lunch time meeting with free lunch as well.”	Physician C
	Workload and staffing levels	“If you’re going to be short staffed starting off in the day there’s absolutely no way anybody can go.”	Nurse/Midwife B
		“people like me who are floaters around the place and can leave there, get up and leave and it’s the people at the bedside that can’t get up and leave and attend these meetings. I see that a lot”	Nurse/Midwife F
		“there is always the potential that you’re going to be called away from some task to do another task that’s considered more important. And we run an acute service here so it’s an acute delivery service and acute neonatal unit.. .. .. So it is difficult for us to get protected time to do things. We don’t have it basically.”	Physician D
		“I know it’s not easy because of staff constraints at the minute. That a lot of leave, not being replaced, and all that, that it is difficult to release people, even for their mandatory training. And therefore, they find it very difficult to come to other training.”	Nurse/Midwife A
	Organisational climate	“I do find it very challenging here to be honest. I organised a talk last week and I had 2 people attend and it was announced by, you know it was very pertinent to everything.”	Nurse/Midwife G
Social context	Interprofessional and multiple disciplinary approach	“the multidisciplinary approach that everybody was involved in it, you know, we can be very segregated. So I think it was important that everybody worked together.”	Nurse/Midwife D
		“I thought this was a good one. Because it brought together the obs [obstetric] and the neonatal end of things. So that was certainly very positive.”	Physician D
		“I think the multidisciplinary aspect of it. I think it wasn’t just one particular person presenting the whole thing. Having a team and each person having their specific work designated.”	Physician E
	Influence of senior staff	“. .. the consultants needing to attend and show the interest. Because here nothing happens unless they show that (. ..) if the consultants support it, certainly they’d get all their Regs [Registrars] and SHOs on board because they’ll do anything that they tell them. And from a midwifery perspective if the managers are on board and encouraging. I think that’s the main thing.”	Nurse/Midwife G
		“And it’s also I think really important as a [high level staff member] to attend these meetings. So I think you set a good example then to the junior staff that these are important to attend.”	Physician E
Individual level factors	Perceived benefit	“But it may be down the road where I’m not chasing every study opportunity that I get, that I would be more selective about topics but it wasn’t an issue for me, the topics, they were all of interest so far.”	Nurse/Midwife E
		“But again it’s very hard to get people who have been, you know a role, an active role in the hospital to take an hour out of their day to attend something, you know unless there’s some carrot there, there was the education bit, there was lunch and it was well advertised.”	Physician D
		“it’s just a suggestion for one thing. Like providing you know, CPD hours for these activities, would make them even more. Would make people more like want to come even more.”	Physician F
		“I loved getting certificates (inaudible speech & laughter), we do have to kind of show that we’re improving our practice and going to different study days (. ..) it’s a good way of bringing the current evidence I suppose into practice. You know and just looking at our own practice and seeing if there’s ways of improving it or not.”	Nurse/Midwife C
		“. . .when people do get certificates they, it does motivate attendance. Because then they can claim that they had one hour at this meeting and they have the certificate then to support that and back them up.”	Physician E

### Barriers and facilitators to presenting

This domain included two themes of individual level factors, and departmental context and resources. The perceived benefit of taking part and having an interest in the topic or format facilitated presenting at Evidence Rounds. Presenting was considered as a more active way to engage with the literature. Some participants had a long-standing interest in their topic and viewed Evidence Rounds as a platform to promote discussion with colleagues. One participant took part because they wanted to experience giving a presentation in an alternative format to a journal club. Another participant shared that recruiting presenters was a continuing problem.

Staffing issues also influenced decisions to present at Evidence Rounds. Even though evidence was presented by 3 HCPs per session, a lot of preparatory work was required from each individual. Another important finding was that some staff were motivated by a strong interest in the topic, a need to set an example for less experienced HCPs or the desire to experience presenting in this unique initiative. Our study found multi-dimensional factors that can be both barriers and enablers to different individuals, at different times and under different circumstances. For example, the level of self-confidence in presenting in front of others could either encourage or discourage potential presenters from taking part.

Health care professionals who saw themselves or others as being deficient in knowledge, skills, or education or those without a research background, identified this as a barrier to presenting. For some participants, their taking part was done to motivate others and learn the process so that they could provide assistance to future presenters. Others presented because they were well-versed on the topic and felt confident to present. One participant mentioned their fear of being asked difficult questions by attendees but chose to present regardless.

The structure of Evidence Rounds whereby three HCPs presented at each session was encouraging for some staff. Some topics can cause information overload if there is a lot of published evidence so sharing the literature and the workload with colleagues helped to minimise any negative impact on work-life balance. Nevertheless, some HCPs viewed their busy schedules and the extra work associated with presenting as barriers.

The transience of junior medical staff was identified as a barrier because they were rotated to different hospital departments or hospitals every 6 months. They were perceived as being less willing to take part because they would be moved soon afterwards. Support from line managers i.e. protected time to prepare for their presentation, was identified as a determinant that would encourage staff to present Table 2.

**Table 2:** Themes and sub-themes likely to explain responses to questions about the barriers and facilitators to presenting

Theme	Sub-theme	Sample Quote	Participant
Individual level factors	Perceived benefit and interest in topic and format	“I think by doing, taking the extra step by presenting you’re learning things as well rather than just sitting down listening to somebody else talk about something. I think if you’re a presenter you would learn more basically and probably benefits you more because you’re taking in all the information.”	Nurse/Midwife C
		“my topic was. .. . which I’m passionate about”	Nurse/Midwife G
		“And I thought that afterwards. I said some people here have no interest in what I’m saying. But I have an interest in doing it.”	Nurse/Midwife F
		“I’ve a big interest in that topic. And also there was concerns raised clinically about. .. .. So I thought here goes, here’s the big opportunity and I’m glad I did it.”	Nurse/Midwife A
		“I think when I read that, you know title, Evidence Round, I feel like it’s a bit different, which I already presented like journal clubs, case presentations and thing like that. So that appealed to me, like you know, should I try something different?”	Physician A
		“I guess there was always a bit of difficulty with picking people who would do the stuff and that will always be a problem. And I’m not sure of what a better way to do that is.”	Physician D
	Self-perceived knowledge and skills	“. .. I’m not too sure that every midwife would be happy to participate. And that kind of worries me a bit because this is supposed to be every man’s or every woman’s kind of, all of our forum. And I’m not too sure if someone who wasn’t that confident, like I’d present a good bit.. .. and I found it quite nerve-wracking. And that was with a lot of support. And that’s just me, I just would feel, like if I was doing it again I probably wouldn’t be as nervous but, or maybe I would. But I’m not too sure how other midwives that hadn’t the same kind of background as myself would feel and that’s the only worrying bit about it.”	Nurse/Midwife G
		“I felt I’m fairly up to date myself with the topic. .. . therefore that didn’t inhibit me to present to the greater group.”	Nurse/Midwife A
		“if they don’t have a background in research or anything, I think it would be difficult to be involved.. . you need to have a little bit of knowledge and background to be able to do that in a competent, confident kind of manner. .. . So I think. .. . their educational status as well would kind of come into play.”	Nurse/Midwife G
		“I don’t think I could see me doing it, no I would not be able to stand up in front and present. Even though I do teach a course. .. . even just when I was sitting there I said "oh there’s no way I would be able to stand up there and do that".. .. . I don’t think I’d have the skills to do it. I wouldn’t be really proficient with you know, the technology”	Nurse/Midwife D
		“I was really worried about, you know the questioning and would I be able to manage the questioning, that was my concern really”	Nurse/Midwife G
	Setting an example	“So I thought I just can’t do it unless I’ve done it and understand it completely. So it was kind of just to get a real insight into the process and to be able to support others.”	Nurse/Midwife G
Departmental context and resources	Workload and staffing levels	“Because when you’re working in a clinical job and you’re trying to keep up to date with research and having to go through an abundance of papers and meta-analysis and research and reviews. It can be very time-consuming. And particularly when you’ve got life outside of work as well [. ..] dividing that work load up between people works really well.”	Physician E
		“And you can see the difficulty in trying to get the volunteers to kind of do the work. Because its work for them, you know I mean there is an effort required. And you know, they already have plenty of work to do. And then you know, this is an additional task for everybody. So it is a challenge to keep things like this going, yeah.”	Physician D
		“You know it isn’t as simple as going in and looking at a journal and kind of looking at the evidence and that, like there’s a lot more work involved [. ..] to be given time. .. .. I think would be important from an organisational perspective.”	Nurse/Midwife G
		“. .. at this level of training you don’t need months you don’t need months to prepare. Once you have the articles, a couple of days. Anything else is excuse.”	Physician C
	Transience of medical staff	“these things work for permanent staff. They don’t work well when staff are coming and going. And that’s again you know the basis of the difficulty with trying to get the medical people to engage in anything. It’s because they are temporary, they’re gone in 6 months’ time, it doesn’t matter really, you know.”	Physician D
	Buy-in from senior staff	“But I do think for somebody on the wards based, I think it would be really important that their managers would be on board and they’d be given time and support to prepare for it. And I think that would be crucial [. ..] if my boss didn’t support it, if she wasn’t, if she didn’t have the buy in or the belief in this. Then you know, that might have been difficult.”	Nurse/Midwife G

### Organisational readiness for change

This domain included two themes of acceptability and appropriateness, and pushing and changing slowly. All participants viewed Evidence Rounds as having a positive impact on their practice and education. It highlighted the need to improve their communication with colleagues in relation to approaches to care delivery. Evidence Rounds helped to ensure practice was based on research evidence as well as their own clinical experience and that of their colleagues. The initiative was acknowledged as having a wider scope, decreased risk of bias and more applicability to decision-making for clinical care than traditional journal clubs. Participants welcomed the opportunity for interprofessional collaboration across multiple professions and disciplines and saw this as a means to network and discuss key issues with colleagues they might work with infrequently. There was recognition that getting together for Evidence Rounds sessions helped to foster links between the midwifery, obstetric and neonatal departments.

Most participants acknowledged that key research findings highlighted as actions from Evidence Rounds were slow to be implemented although some recommendations had been implemented in practice. Bridging the gap between research and practice is often contingent on additional steps. Evidence Rounds was seen as a platform to begin a conversation and start to plan the formal process of updating or creating new guidelines so that there could be a widespread change in practice. One participant noted that having the relevant guideline developer in attendance would increase the likelihood of getting the evidence into practice Table 3.

**Table 3:** Themes and sub-themes likely to explain responses to questions about organisational readiness for change

Theme	Sub-theme	Sample quote	Participant
Acceptability and appropriateness	Impact on practice and education	“Seriously, I think it’s one of the best things that’s happened in a long time for advancing our practice and education.”	Nurse/Midwife D
		“. .. the last meeting, it raised a lot of questioning. So and we all think we are all doing the same thing. But the last meeting showed that we don’t really do the same thing.”	Physician C
		“Evidence Rounds were very, I think concise. And all the documents were there. I think it gives you a much better overview of of [sic] things. And it certainly has led us to question our practice. And the one thing that jumps to mind was the medication pre-intubation. [. ..] Evidence Rounds are very good at making us all think about our practice. And how we can improve it. Are we doing things safely? Are we in keeping with national and international evidence supported best practice, recommendations?”	Physician E
		“I don’t want to use the word ignorance but it definitely educates people into, you know. .. .[trails off]. Again, a flaw of medical practice is the kind of folklore of practice. That people work in one hospital and oh they all did this here and that’s why we’re doing it now. Why aren’t you doing this, because they’re all doing that there? But people often fail to look at what the evidence is to support the treatment or to support the practice.”	Physician D
	Comparison with journal club	“journal clubs are good if they’re used the right way. But what happens an awful lot is that people focus on one article. And it may not be the most up to date article. And it’s just one particular aspect. Whereas the Evidence Rounds I find are really good because it’s more like you’re going to all the various repositories, to access your evidence. You’re looking at your Cochrane review and your meta-analysis. You’re getting more of a, I guess, an eagle view of it.”	Physician E
		“. .. . journal club tends to just whip out one article. .. . and often it may have a biased view. .. .”	Physician D
		“I know we used to have a journal club. .. that went for a while but it didn’t take off.”	Nurse/Midwife C
	Promoting interprofessional collaboration across multiple disciplines	“it’s a platform for different groups [to] say, do we agree with it, do we not agree?”	Physician C
		“I liked the multidisciplinary approach, I thought that was brilliant. I really and I loved the fact that so many of the midwives even came from the other wards that I wouldn’t know very well. And they participated and asked questions. .. you got a great discussion going.”	Nurse/Midwife E
		“I think it’s very, very important here. .. . that it is very much combined obstetrics and neonates [. ..] [midwives] need to be able to speak at meetings and in groups and kind of, because [they] do have such a different perspective. But this has never been encouraged really in the Irish setting.”	Nurse/Midwife G
Pushing and changing slowly	Implementation of evidence	“.. . some things are not needed to go in the guidelines but again it takes time for anything to change. But again I think it doesn’t matter, it’s important to talk about it and to, because things like this are pushing and changing slowly.”	Physician B
		“We have changed practice, we can see it already.”	Nurse/Midwife F
		“And that’s definitely changed practice because now we are bringing it in [Evidence Rounds session on Timing of umbilical cord clamping] [.. .] And we’re discussing it and we’re aware of it [. ..] And it’s coming on the new neonatal guidelines so that’s going to be, it was great to have that evidence to know whether we wanted it or not [. ..] The progress and the changes will be slow but the awareness is there, it’s just sitting down to actually get the work done.”	Nurse/Midwife E
		“I think it gives you idea to, you know, change the practice but it will not straight away. .. . once you. .. have some audit or something because we would change the practice.. . So that Evidence Round will give you a thought and then you can take that point and then you can. .. change the the recommendation and the practice”	Physician A
		“we haven’t changed too much.”	Physician E
	Writing and updating clinical practice guidelines	“you can’t just change practice after an Evidence Round, it has to be put into a guideline before we can, like we can’t just say oh we’re going to use this drug, that drug and then do it, we actually have to have it in the guidelines.. .. It’s gonna [sic] take time to do the guideline out and you know they have to go to guideline meetings then and then after that, it will be put into practice. So it’s not going to be overnight that the practice will be changed. But it will be eventually.”	Nurse/Midwife C
		“people setting the guideline for the hospital are the one who should really attend. Otherwise we would just be speaking about the evidence without applying it to our daily practice.”	Physician F

### Communication and dissemination of information

This domain included two themes; modes of delivery and communication and dissemination strategy considerations. We asked participants questions to gain insight into their preferred modes of delivery when receiving communications and disseminated information. One important finding is that participant feedback did not identify a single mode of delivery of information that would have engaged all staff. Therefore, our decision to employ a multi-component strategy was appropriate for our target population despite a lack of evidence that this is the most effective approach [27, 28]. HCPs agreed that posters displayed in appropriate hospital areas were effective at drawing attention to upcoming sessions. The use of email to communicate information about Evidence Rounds elicited diverse responses from participants. For individuals who spent at least part of their working day with access to a computer or mobile phone and had a work email address, this was a convenient way to receive information. However, it was not an effective way to reach others such as staff midwives who were more clinically based and either were not issued with, or did not regularly access their professional email accounts. Not all participants used the Evidence Rounds website but those who did, found it accessible and an easy way to retrieve and refer others to past presentations. One participant found the critical appraisal tool links useful to prepare for their presentation. For one healthcare professional who limited their engagement with technology, the website was not a suitable medium. Participants had mixed opinions about the use of text messaging and other mobile messaging technologies such as WhatsApp. On one hand, they acknowledged that they were a means whereby information could be communicated to the intended receiver in an easy and direct manner. On the other hand, many staff voiced concerns that work-life boundaries might be violated or feared that they might be bombarded with messages particularly when they were not working. Many of the HCPs were involved in shift work, which compounded their concern regarding this issue. Word of mouth was a popular method among staff to encourage their colleagues to attend sessions. Multiple reminders and reminders on the day of the sessions were viewed as having a particularly positive impact on attendance Table 4.

**Table 4:** Themes and sub-themes likely to explain responses to questions about communication and dissemination of information

Theme	Sub-theme	Sample Quote	Participant
Modes of delivery	Posters	“The laminated posters definitely for me were excellent because when you’re busy in the clinical area they stand out on a notice board to you.”	Nurse/Midwife B
		“the posters are good as well. A lot of people are very visual in terms of taking in information. And if they can see something they go, oh right okay yeah, yeah I must remember to go to that meeting. I think a printed sign is useless. I think you need to have some kind of a picture on it. Because people are drawn to images and pictures and bright colours.”	Physician E
	Email	“it’s different if, for some staff like us, we’re emailing all the time with work so our emails are coming through to our mobile phone. But the nurses that wouldn’t be the case, they wouldn’t have personal emails for work. So they’re not going to get them.”	Nurse/Midwife E
		“I think email is probably the number one way of communication with people nowadays.”	Physician D
		“it’s trying to reach them appropriately because emails. .. . to the wards only goes to the managers. So it’s how good they are at sharing information and or prioritising it”	Nurse/Midwife G
	Website	“was great and it’s very, it’s lovely, it’s a very easy to use and easy to navigate website so yeah I found it useful (. ..) it was good to see the other talks and I kind of would just have a little look through again.”	Nurse/Midwife G
		“I think it’s a really good go-to place, a good repository then just to access the information.”	Physician E
		“I think it’s fantastic what you put about analysing articles. .. I would think you know oh my god, what is this, what is important in this or not? So I think this is very, very nice and useful that you put it that way yeah.”	Physician B
		“I would not be a typical person. .. because I am not really the most enthusiastic researcher.”	Physician C
	Word of mouth	“I think to be honest for me as a practitioner trying to encourage people to attend, going around on the day and reminding people was the thing that actually worked the best.”	Nurse/Midwife G
	Mobile technologies and work life boundaries	“one of our professional issues we set for ourselves is no use of mobile phones in the work place.”	Nurse/Midwife B
		“I think there needs to be very strict boundaries within which WhatsApp would work. I think your group is going to constantly change. So you may be missing out people who would otherwise attend it. You may be constantly texting people who may need to be removed from the group [.. .] some people get very annoyed if they’re on a day off, or if they’re not working a shift and they’re constantly getting these alerts.”	Physician E
		“you have to be very careful or they’ll opt out very quickly.” [WhatsApp groups]	Nurse/Midwife E
Communication and dissemination strategy considerations	Multiple reminders	“unfortunately, some medical people, like kids you really have to push them and nag them to get something, you know. .. .. who is going to present? Who is going to present? Who is coming? Who is coming?”	Physician C
		“And we kept, at any meetings we had we kept saying the next topic is on. .. . make sure you’re on that day. And in the morning beforehand, going around saying 'Don’t forget now, make sure you go to that today'.”	Nurse/Midwife A
	Organisational issues	“that’s one of the great challenges, it is, within our organisation, to try and share information.”	Nurse/Midwife G
	Multiple formats	“I think you can’t really do it just one particular way.”	Physician E

### Sustainability

Finally, we asked HCPs about their perceptions of the sustainability of Evidence Rounds and how they would make future initiatives more sustainable. Sustainability is difficult to measure [29] so our qualitative approach allowed us to gain an understanding of context to help others select appropriate strategies during implementation to improve sustainability.

This domain included two themes; staff engagement and collaboration and individual and departmental influences on sustainability. Perhaps the most striking finding is the influence of resources and particularly the HCPs themselves, on sustainability. Their availability, schedules and workloads, level of interest and motivation, the engagement of senior-level staff and their willingness to lead and become champions for initiatives were hugely important factors. These results corroborate suggestions that behaviour change theory may be useful to positively impact implementation processes. HCPs identified a number of factors key to the sustainability of Evidence Rounds and similar initiatives after the support of the KT professional would be terminated. Staff representatives from both the neonatal and obstetric units would need to take ownership and assume responsibility for administrative tasks such as planning and scheduling the meetings. Some participants viewed champions as essential for sustainability. Two participants believed that there needed to be a dedicated person whose job it was to oversee education and another thought their role should include developing clinical practice guidelines.

All participants remarked positively on either or both of the interprofessional and multiple disciplinary aspects of the initiative. One individual believed that senior level staff from one discipline were more invested in keeping it going than those from the other discipline and worried about the impact of this. There was a sense that it was not always easy to come up with topics of simultaneous interest to midwifery, neonatal and obstetric departments. Evidence Rounds was just one of many educational opportunities open to staff during their working week. Taking into consideration the already busy workloads of the healthcare professionals, it was not easy to find staff to volunteer to take on the extra responsibility required to keep it going. Buy-in from senior level staff and having a consultant run the sessions were considered factors that might encourage staff to attend. Rotating presenters and dividing tasks between a team of three was a means of keeping the workload associated with presenting at a manageable level.

Assigning a HCP to pre-schedule the sessions for the coming year was suggested by multiple participants. Participants mentioned the need to involve someone with experience in performing systematic literature searches and to provide additional support to upcoming presenters Table 5.

**Table 5:** Themes likely to explain responses to a question about the sustainability of Evidence Rounds

Theme	Sub-theme	Sample quote	Participant
Staff engagement and collaboration	Need for opinion leaders and champions	“it needs to have an obstetric lead and a neonatal lead. I think it really needs both of them.”	Nurse/Midwife G
		“it would up to them to organise with. .. the junior doctors and the nursing staff as well. That they would have to participate at some point in time. And so getting people, but you do need a designated go-to person in that particular area. To design the scheduled meetings and to fix them in the calendar.”	Physician E
		“. .. we need to have somebody, in my opinion, whose total, total role is looking at evidence and guidelines and producing that so that practitioners can change practice or you know develop guidelines for practice.”	Nurse/Midwife A
		“if you have people whose job, whose professional role is to provide education, it works well. We are lacking that type of person on our end of things. So that’s why they often, these things run for a period of time and then they just kind of fall apart.”	Physician D
		“It needs a leader.. .. . to push it and to support each time. And to do the searches and to support the staff.”	Nurse/Midwife G
	Buy in from senior level staff	“I’m not too sure that they’re attending or they understand the importance or they’re kind of, that the managers kind of see it as an important process (.. .) It really needs to start the high up. And if we could get the buy in from both of them and then they encouraged their teams, it would certainly be a lot more effective.”	Nurse/Midwife G
		“If it’s run by the consultant, people would attend even more.”	Physician F
	Interprofessional and multiple disciplinary approach	“the biggest thing I got out of it was the multi-professional involvement because we do a lot of our training and updating ourselves in separate capacities, even though yet we work together to care for the woman, the one woman in front of us. But we’re coming at different angles all the time. So I think it’s hugely important to bring it forward and even incorporate it in more and more of our training. That we’re working together, we’re updating our skills together, we’re training together. And as a result, we’re caring for the woman together.”	Nurse/Midwife B
		“And it was such an involved group as well, you know a diverse group. Usually when we’d have something, it might be just the nurses that are there, everybody was attending.. .. The CNMs, the nurses, the doctors, the regs, so I thought that was good.”	Nurse/Midwife D
		“I feel that’s because none of the consultants from [department X] got really behind it. And I think the [department Y) would be quite happy to take it as their baby and run with it basically. And that’s a huge problem, that would be a huge problem because there is no baby without having all the services involved.”	Nurse/Midwife G
		“The only problem is to find a common topic with the obstetricians.”	Physician C
Individual and departmental influences on sustainability	Skills and knowledge to access evidence	“if you haven’t got help with someone doing the literature searching that’s a lot that’s a big part of the work, so to try and get that done every month will be hard, on our own.”	Nurse/Midwife E
		“you had researched the papers and given them to them beforehand. That was good as well. I think it made the, their job a little bit easier. But also my question would be if they were presenting Evidence Rounds in the way they were presented, would they have known where to go to access these papers that you gave them? Or would they have known how to access them? So if individuals were left to their own devices to carry on with Evidence Rounds. Without the various reviews being supplied to them, I don’t know that they would actually know where to go to. And maybe I’m completely wrong. You might get a more limited number of articles presented.”	Physician E
	Competing with clinical workload and other educational sessions	“And you can see the difficulty in trying to get the volunteers to kind of do the work. Because its work for them, you know I mean there is an effort required. And you know they already have plenty of work to do. And then you know this is an additional task for everybody (.. .) there are so many education sessions, it’s very difficult to you know squeeze another one in. So you know it’s a challenge I think just to keep people interested and keep them going, yeah hard work.”	Physician D
		“the [department z] site has few people turn up, it’s also they have their Friday lunch time meeting with free lunch as well.”	Physician C
	Maintaining interest and subject saturation	“the enthusiasm for these things wax and wane depending on who the staff are. And then you run out of topics to some degree as well. You know you do all the good ones and the big ones initially. And then as time goes on then people are really scraping the barrel to look for things.”	Physician D
		“I thought what worked well was when we, at the very end we were very clear, from the get go that we said at the end of this we want to have presenters for the next rounds and have decided a topic. I think leaving it creates just too much space. And unless you get people to commit. I think that just doesn’t work great.”	Nurse/Midwife G
		“I think new projects are always great. Sustainability is one of the big problems. And keeping people motivated.”	Physician E
	Rotation of presenters	“I think if it was the same people presenting all the time, it would be a lot of work on the same people. If it was divided up equally then I think it would be good.”	Nurse/Midwife C
		“if it was again, like a rotation. .. And it should alternate and people have to do it. That will make it I think more regular and people probably will have to do it. It’s not an optional thing, it’s a mandatory thing.”	Physician F
	Scheduling and frequency	“there’s a schedule and there’s time frames for people to meet, I think once that’s written into the yearly schedule of events, I think that people will participate in it”	Nurse/Midwife A
		“Yeah, probably doesn’t need to be every month [. ..] we will run out of topics at this tempo”	Nurse/Midwife E

## Discussion

This study sought to identify the barriers and facilitators to attending and presenting at Evidence Rounds. Our findings agreed with evidence from other studies that the provision of refreshments may be associated with increased HCP attendance at educational events [30, 31].

We wanted to improve our understanding of HCPs’ perspectives of Evidence Rounds as a dissemination strategy. We asked multiple questions to gain insight into their preferred modes of delivery when receiving communication and disseminated information.

Overall, our study findings were consistent with a mixed methods study also conducted in Ireland, to reach consensus on priorities for clinical learning environments for postgraduate medical education [32]. Among the most important domains identified by participants in that study were: support for residents; opportunities to learn with senior doctors; engagement in clinical teams; organisational and working conditions.

### Strengths and limitations

Evidence Rounds was an example of pragmatic, community-engaged dissemination and implementation research [33, 34] in which the community is the target population of HCPs. It came about because key stakeholders within our target audience approached staff at the National University of Ireland, Galway having identified a need for support in translating research evidence into practice. One of our authors (AC) was recruited as a PhD student to take on this project as a part of her PhD research, having had experience of implementing Evidence in Practice Groups with HCPs as part of CEBIS in the UK. The key strength of this study is the rich data from our focus groups and interviews, which provides context and contributes to the development of evidence about HCP perspectives on the implementation of KT strategies. Research has consistently shown that contextual factors in a given setting play a large role in the success or failure of these types of activities. We employed qualitative methods of research as a means to gain understanding of interactions between individuals, organisations and their unique contexts [35]. The key finding of studies that have undertaken process evaluations is not only the significance of contextual factors but the fact that they can often have the most significant impact on the intervention [14]. This information could be used to generate insights that decision-makers can use to plan, develop and implement their own initiatives. Notwithstanding, this study has some limitations. Despite our best efforts, recruitment of participants was low. Several factors could have attributed to this for example, the department where most staff worked was above capacity during the period when the focus groups were held. Nevertheless, one-to-one interviews were offered as an alternative.

It is not clear whether our participants were a representative sample of the population. More than half had presented or were involved in the co-design or implementation of the initiative. Therefore, this group may be more invested in Evidence Rounds than other potential participants. We did not capture the perspectives of healthcare professionals who did not attend Evidence Rounds. The inclusion criteria for our study specified that participants must have attended at least one group session.

Another limitation of the study is that the main researcher who implemented the initiative also moderated the focus groups and interviews and was involved in analysis and interpretation. Participants may have felt reluctant to share negative perceptions. To address this concern, at the start of each interview or focus group we emphasised that both positive and negative feedback was being sought to continue the initiative and make it more effective or to make recommendations for future initiatives.

In one systematic literature review, the authors reported that a timeframe of two or more years is required to examine the sustainability of evidence based interventions [22]. Tricco and colleagues (2016) reported that the KT interventions included in their scoping review focused on sustainability ranged from 61 to 522 weeks [23]. Our initiative was implemented over 9 months so this timeframe may not be adequate for optimal conditions to ask participants questions about sustainability.

Framework analysis uses an applied rather than a theoretical approach to research [25]. Therefore, another potential limitation of our study is the lack of theory in our investigation of barriers. The use of a validated tool such as the Theoretical Domains Framework [36–38] would have allowed us to map our barriers to pre-specified behaviour change domains.

### Implications for research and practice

Further research might explore how to leverage social media platforms to effectively communicate and disseminate evidence to a targeted population. Evidence Rounds was an initiative for HCPs in Ireland, which is classified as a high income country [39]. Questions remain as to how the perspectives of health care professionals working in low and middle income countries might differ from those of our participants. Another important issue for future research is to determine how to integrate the values and preferences of patients, carers or the public, into initiatives like Evidence Rounds to inform and improve the decision-making process [40]. Further, our findings may have implications for the understanding of how behaviour change theory might be used to optimise initiatives and strengthen capacity to improve the implementation of evidence.

The findings of this study uncovered a number of important points to inform individuals planning, developing or implementing initiatives aimed at HCPs. We encourage others to consider interprofessional and multiple professional/disciplinary platforms for these types of initiatives as this approach was valued highly by staff. Those planning similar initiatives may consider multi-component strategies. Our HCPs found more benefit relating to the provision of patient care in group sessions focusing on the best available evidence than on previous events like journal club which critically appraised single articles. Effective communication and dissemination aimed at HCPs requires careful consideration of a number of factors including the mode of delivery, scheduling, frequency, and organisational, departmental and individual-level preferences. Feedback during implementation from the target population may guide decisions to maintain, remove or modify aspects of the strategy. Others implementing similar initiatives may consider factoring in the provision of support and training for presenters who need help with critical appraisal, data presentation, statistical inference etc. The development of a plan for presenters and attendees would be ideal to build organisational capacity. Our health service staff did not feel that they had the skills to perform adequate searches on clinical topics or questions. Like other authors, we recommend the involvement of information specialists, librarians or individuals with experience of designing and conducting search strategies [41]. We also recommend involving and collaborating with guideline developers to increase the likelihood of implementation of research findings.

## Conclusions

We set out to identify barriers and facilitators to attending and presenting at group sessions from the perspectives of HCPs, to gain an understanding of their views of Evidence Rounds as a dissemination strategy and to generate insights to improve the sustainability of future initiatives. The results of this study and our analysis have extended our understanding and may be useful for guiding the development and implementation of future KT strategies for HCPs. Our focus groups and interviews highlighted the variability in preferences of mode of delivery in our target audience suggesting the multi-component approaches can be useful. They helped us gain insight into the influence of organisational and individual level factors (e.g. buy-in and support from senior staff, staffing levels and scheduling, self-confidence) on the willingness and ability of HCPs to attend, present at and sustain these types of initiatives.

Although HCPs invariably work in complex systems with unique contexts, this paper may help others to understand factors that can impact the implementation of initiatives to disseminate key research findings and promote evidence informed practice.

## Additional files

Additional file 1: FGI Guide_BMC Med Ed. (PDF 261 kb)

Additional file 2: Informed consent_BMC Med Ed. (PDF 140 kb)

## Abbreviations

AC: Aislinn Conway
CEBIS: Clinical Evidence Based Information Service
CREC: Clinical Research Ethics Committee
DD: Declan Devane
GA: Gestational age
HCP: Healthcare professional
HRB-TMRN: Health Research Board Trials Methodology Research Network
KT: Knowledge translation
MD: Maura Dowling
NCHD: Non-consultant hospital doctor
NMPDU: Nursing Midwifery Planning and Development Unit
PPROM: Preterm premature rupture of the membranes
