# Supplementary material for: Implementing an initiative promote evidence-informed practice: part 2—healthcare professionals’ perspectives of the evidence rounds programme
Source: BMC Med Educ. 2019 Mar 6;19:75. doi: 10.1186/s12909-019-1488-z (PMC6402168; doi:10.1186/s12909-019-1488-z)
Supplement: Supplementary file 1 — FGI Guide_BMC Med Ed. (PDF 261 kb) [file 12909_2019_1488_MOESM1_ESM.pdf]

Evidence Rounds

Focus Group and Interview Guide

Topic	Question
Introduction	1. Can you please introduce yourself and state your job title and department?
Barriers and facilitators to attending and presenting at Evidence Rounds	*Attendance:* You have attended at least one Evidence Rounds session.<br>2. Why did you decide to attend?<br>3. What factors have enabled/would enable you to attend?<br>4. Certificates of attendance / participation were requested by staff and have been sent to those who attended the last 3 sessions. Do you think they may motivate staff to attend?<br>5. Lunch from Mr Waffle is provided at each session. What do you think about this?<br>6. What factors have prevented/would prevent you from attending?<br><br>*Participation (presenting):*<br>7. Why did you decide to present?<br>or<br>What has prevented you from presenting?
Sustainability<br><br>Capacity building	The next Evidence Rounds session, will be the sixth and final one that I will be working on.<br>8. Would you like to see it continue into the future?<br><br>9. If so, what do you think will have to happen for it to be delivered over the long-term?<br><br>10. If not, and we asked you to design it, what would you like to have instead?
Dissemination goal 1: Increasing the *reach* to a variety of audiences	11. How did you first hear about Evidence Rounds?<br><br>12. Are you aware of the dedicated Evidence Rounds website?<br><br>13. If so, have you visited it and what do you think about it?<br><br>As well as the website, I also communicated and disseminated information and evidence to you using:<br>• email reminders from your colleagues

Topic	Question
	• additional emails from myself<br>• posters hung in staff areas to notify staff of upcoming sessions<br>• a desktop shortcut to the website has been added to the pc in the neonatal unit<br>• Social media accounts on Facebook and Twitter<br><br>14. How did you find these?<br><br>15. Which was most/least useful?<br><br>16. Can you suggest other methods that might be useful to you?<br><br>17. Other staff have suggested using WhatsApp groups or webtext. What do you think about these?
Dissemination goal 2: Increasing *motivation* to use and apply the information	18. What do you think of Evidence Rounds and applying evidence to your practice?
Dissemination goal 3: Increasing the *ability* to use and apply the evidence	19. Do you think initiatives like Evidence Rounds increase your ability to use and apply the evidence? If yes, what specifically? If not, why not?
Ending	20. Overall, what do you think worked well in relation to Evidence Rounds and what might be improved for future initiatives?
