# Supplementary material for: Implementing an initiative promote evidence-informed practice: part 2—healthcare professionals’ perspectives of the evidence rounds programme
Source: BMC Med Educ. 2019 Mar 6;19:75. doi: 10.1186/s12909-019-1488-z (PMC6402168; doi:10.1186/s12909-019-1488-z)
Supplement: Supplementary file 2 — Informed consent_BMC Med Ed. (PDF 140 kb) [file 12909_2019_1488_MOESM2_ESM.pdf]

# INFORMED CONSENT FORM FOR HEALTH CARE PROFESSIONALS

This informed consent form is for health care professionals (HCPs) at University Hospital Galway who are invited to participate in the following study:

| | |
|---|---|
| Study Title | Evidence Rounds: a targeted initiative to disseminate research evidence to health care professionals |
| Researchers | Aislinn Conway & Professor Declan Devane |
| Funding/Sponsorship: | • Nursing & Midwifery Planning & Development Unit (NMPDNU) Nursing and Midwifery Innovation Initiatives Funding<br>• Health Research Board Trials Methodology Research Network<br>• National University of Ireland Galway |

This Informed Consent Form has two parts:
- Participant Information Leaflet (containing details of the study)
- Participant Consent Form (to sign if you choose to participate)

## Part I: Participant Information Leaflet

***Introduction***

**Title of study**
Evidence Rounds: a targeted initiative to disseminate research evidence to health care professionals

**Study objective**
This study aims to improve the understanding of determinants as experienced by health care professionals (HCPs) in relation to attending and participating in initiatives such as Evidence Rounds.

**Invitation to take part in the study**
You are being invited to participate in a research study. Before you decide, it is important for you to understand why the research is being done and what it will involve. This Participant Information Leaflet will inform you about the purpose, and the potential risks and benefits of the study. If you agree to participate you will be required to sign a Participant Consent Form. If you do not understand any of the words or concepts used, please let us know and we will be happy to make clarifications or answer questions at anytime.

***Purpose of the research***

**Research Question**
What are the barriers and facilitators experienced by HCPs in relation to attending and participating in targeted educational initiatives to promote evidence informed practice?

**Background**
The focus of dissemination research is on examining strategies used to communicate and spread information to targeted users. In studying Evidence Rounds as a multi-component dissemination strategy, this doctoral project aims to examine the process of designing, implementing and developing an initiative targeted at health care professionals to promote and facilitate evidence informed practice at University Hospital Galway (UHG). We want to learn more about the barriers and facilitators to initiatives such as Evidence Rounds experienced by you so that we can have a greater understanding of how to design and implement successful initiatives in the future. Your participation is likely to help us find out more about how to implement initiatives to communicate and disseminate research evidence to HCPs.

**Participant Selection**
You are being invited to take part in this research study because you have attended at least one Evidence Rounds session. We are asking you to help us learn more about communicating and disseminating evidence to health care professionals. Your experience as a health care professional can contribute much to our understanding and knowledge of implementing initiatives to reduce the knowledge to action gap and promote evidence informed practice.

**Duration of Study:**
This research will involve your participation in a focus group and/or an interview each

of which may last up to one hour.

**Participation- what it involves**
Your participation in this research is entirely voluntary. You should only take part in this research if you do wish to do so, and choosing to refuse participation will not lead to any negative consequences or have any bearing on work-related evaluations or reports. You may stop participating in the focus group/interview at any time.

If you accept our invitation, you will be provided with a copy of this information leaflet to read and you will be asked to sign a Participant Consent Form. We will contact you to arrange a suitable time, date and location for a one to one interview and/or a focus group with 2-8 other professionals. Aislinn Conway and a moderator will guide this discussion. We will start by making sure that you are comfortable with participation. At this point, we can also answer questions or provide clarifications about the research.

Principle topics for discussion include: your experience of having attended or taken part in Evidence Rounds and your perceptions of barriers and facilitators to your participation in an initiative like this.

All interviews will be audio-recorded and written notes will be taken. You do not have to share any information that you are not comfortable if you are not comfortable doing so. If you do not wish to answer a question during the discussion, you may say so and the interviewer will move on to the next question. You do not have to give us a reason for not responding to a question, or for refusing to take part. You may change your mind later and stop participating, even if you agreed earlier.

**Reimbursements**
You will not be offered any monetary incentive to take part in this research. We do not anticipate that any reimbursements for expenses incurred as a result of participation in the research will be required.

**Confidentiality**
We will not ask you to share personal and confidential information. The research team will protect your privacy and confidentiality at all stages of the research process. We will not share information about you to anyone outside of the research team. Any information about you will have a number on it instead of your name. Only the researchers will know what your number is and we will ensure that it will not be shared with or given to anyone else. The research findings will be shared more broadly, for example, through publications and conferences. The entire focus group/interview will be audio-recorded. The information recorded is confidential, and no one except the research team and a transcriber will have access. The digital recordings and electronic files will be kept on a password-protected computer in NUI Galway

The following applies if you are participating in a focus group:
We will ask each of you to keep what was said in the group confidential. You should know, however, that we cannot stop or prevent participants who were in the group from sharing things that should be confidential.

**Who to Contact**
If you have any questions, please contact:

Name: Aislinn Conway, PhD Fellow
Address: Health Research Board Trials Methodology Research Network, 1st floor School of Nursing and Midwifery, National University of Ireland, Galway
Phone: 087-3349755

Email: a.conway18@nuigalway.ie

This proposal has been reviewed and approved by Galway Clinical Research Ethics Committee, whose task it is to make sure that research participants are protected.

*Thank you for taking time to read this information leaflet*

## Part II: Participant Consent Form (non-medical research)

Participant Identification Number: ______________________________ (to be completed by researcher)

I have been invited to participate in this study focusing on an initiative to disseminate research evidence to health care professionals.
I have read the participant information leaflet. I have had the opportunity to ask questions about it. Any questions I have asked have been answered to my satisfaction. By providing my details in the table below I am voluntarily consenting to be a participant in this study.

| **Participant Details** | |
|---|---|
| Name (Print) | |
| Signature | |
| Date (dd/mm/yyyy) | |

### Statement by the Researcher/person taking consent

I have provided the information leaflet to the potential participant at least 24 hours prior to seeking consent and to the best of my ability and made sure that the participant understands the study.

I confirm that the participant was given an opportunity to ask questions about the study, and all the questions asked by the participant have been answered correctly and to the best of my ability. I confirm that the individual has not been coerced into giving consent, and the consent has been given freely and voluntarily.

| **Details of Researcher/Person Taking Consent** | |
|---|---|
| Name (Print) | |
| Signature | |
| Date (dd/mm/yyyy) | |
